# Effect of Intraocular Lens Tilt and Decentration on Visual Acuity, Dysphotopsia and Wavefront Aberrations

**DOI:** 10.3390/vision4030041

**Published:** 2020-09-14

**Authors:** Zahra Ashena, Sundas Maqsood, Syed Naqib Ahmed, Mayank A. Nanavaty

**Affiliations:** 1 Sussex Eye Hospital, Brighton & Sussex University Hospitals NHS Trust, Eastern Road, Brighton BN2 5BF, UK; zashena@nhs.net; 2 Eastbourne District General Hospital, Kings Drive, Eastbourne BN21 2UD, UK; sundas.maqsood1@nhs.net; 3 Northampton General Hospital, Cliftonville, Northampton NN1 5BD, UK; syed.ahmed15@nhs.net; 4 Brighton & Sussex Medical School, University of Sussex, Falmer, Brighton BN1 9PX, UK

**Keywords:** intraocular lens, tilt, decentration, wavefront aberration, visual acuity, dysphotopsia

## Abstract

Tilt and decentration of intraocular lenses (IOL) may occur secondary to a complicated cataract surgery or following an uneventful phacoemulsification. Although up to 2–3° tilt and a 0.2–0.3 mm decentration are common and clinically unnoticed for any design of IOL, larger extent of tilt and decentration has a negative impact on the optical performance and subsequently, the patients’ satisfaction. This negative impact does not affect various types of IOLs equally. In this paper we review the methods of measuring IOL tilt and decentration and focus on the effect of IOL tilt and decentration on visual function, in particular visual acuity, dysphotopsia, and wavefront aberrations. Our review found that the methods to measure the IOL displacement have significantly evolved and the available studies have employed different methods in their measurement, while comparability of these methods is questionable. There has been no universal reference point and axis to measure the IOL displacement between different studies. A remarkably high variety and brands of IOLs are used in various studies and occasionally, opposite results are noticed when two different brands of a same design were compared against another IOL design in two studies. We conclude that <5° of inferotemporal tilt is common in both crystalline lenses and IOLs with a correlation between pre- and postoperative lens tilt. IOL tilt has been noticed more frequently with scleral fixated compared with in-the-bag IOLs. IOL decentration has a greater impact than tilt on reduction of visual acuity. There was no correlation between IOL tilt and decentration and dysphotopsia. The advantages of aspheric IOLs are lost when decentration is >0.5 mm. The effect of IOL displacement on visual function is more pronounced in aberration correcting IOLs compared to spherical and standard non-aberration correcting aspherical IOLs and in multifocal versus monofocal IOLs. Internal coma has been frequently associated with IOL tilt and decentration, and this increases with pupil size. There is no correlation between spherical aberration and IOL tilt or decentration. Although IOL tilt produces significant impact on visual outcome in toric IOLs, these lenses are more sensitive to rotation compared to tilt.

## 1. Introduction

Correct alignment of intraocular lens (IOL) is fundamental for optimal visual function following cataract surgery. IOL tilt and decentration have negative impact on visual performance by inducing optical aberrations and, in extreme cases, decreasing visual acuity [1]. IOL tilt is defined as the angle between the IOL axis and the reference axis and IOL decentration is obtained from the distance between the IOL centre and the reference axis. However, there is no unique definition in the literature as different reference axes have been used in different studies. For instance, pupillary axis has been used as the reference axis by several studies in the past [2,3]. However, corneal vertex [4] and visual axis [5] were employed by newer devices and studies to measure the IOL tilt and decentration.

IOL tilt and decentration could be secondary to various aetiologies. The extent and direction of preoperative crystalline lens tilt have been demonstrated to be strongly correlated with postoperative tilt [4,6,7]. Asymmetrical IOL fixation (partly in bag and partly in sulcus) and capsular tear during capsulorrhexis were reported as some causative factors to IOL tilt and decentration [8]. While long axial length, thicker lens, larger capsulorhexis, and severely malformed capsulorhexis were confirmed to increase the risk of IOL decentration, [9,10,11] previous pars plana vitrectomy (PPV) and short axial length had a positive correlation with IOL tilt [11]. IOLs are more prone to tilt in patients with a short axial length. The possible explanation is that the crystalline lens is more likely to tilt in the crowded intraocular space [11]. Nevertheless, following an uneventful cataract surgery, capsular fibrosis is a common cause for tilt and decentration in horizontal and vertical meridians [12]. Ding et al. reported significantly higher risk of IOL decentration in *x*-axis and IOL tilt in eyes with partial IOL optic overlap by the anterior capsule compared to the group with total overlap [13]. IOL displacement can cause defocusing, astigmatism, and wavefront aberrations postoperatively [14,15]. For instance, horizontal tilt of an aspheric IOL induces against-the-rule astigmatism. For 5° and 10°of IOL tilt, the induced astigmatism levels were 0.14 dioptres (D) and 0.56 D for 28 D aspheric IOLs respectively [16].

The effect of IOL tilt and decentration on visual function varies with the design of IOLs. Aspheric IOLs, which are primarily designed to compensate for the corneal positive spherical aberration, are shown to produce more optical quality degradation if tilted or decentred, compared to spherical IOLs [17]. Expectedly, for toric IOLs, tilt and decentration lead to less predictable astigmatism outcomes [16]. The alignment and amount of toricity along with orientation of tilt have resulted in under-correction or over-correction of astigmatism in toric IOLs in theoretical eye models [16]. In multifocal IOLs, decentration might result in a different light distribution between the distance focus and near focus [18]. With regards to IOL design, 1-piece and 3-piece, hydrophobic and hydrophilic IOLs have been reported to perform equally well in terms of decentration and tilt [11,19].

In 1988 Phillips et al. [20] reported a mean tilt of 7.8 ± 3.0° and mean decentration of 0.7 ± 0.3 mm in a group of 14 individuals with in-the-bag IOL. However, luckily this complication has noticeably minimized due to significant advancements in surgical skills and IOL designs [21]. De Castro et al. [2] noticed an average of 2.6° tilt and 0.4 mm decentration in 12 pseudophakic individuals and Rosales et al. [22] detected 1.54° tilt and 0.21 mm decentration in the same number of patients. Interestingly, mirror symmetry has been noticed between the eyes for tilt around the vertical axis and decentration in the horizontal axis [4,6,7,22]. Some studies have evaluated and compared preoperative crystalline lens tilt and decentration with postoperative IOL displacement. On average a 5° inferotemporal tilt was noticed in both crystalline lenses and IOLs, [4,5,6,7] indicating that the fovea was slightly temporal to the pupillary axis of the eye [6]. Furthermore, significant correlations were found between pre- and postoperative data for both tilt and decentration [6,7].

The amount of IOL tilt and decentration that can be tolerated without any detriment to visual outcomes is also studied in the literature. A meta-analysis of clinical studies on IOL tilt and decentration reported that 2–3° tilt and a 0.2–0.3 mm decentration are common and clinically unnoticed for any design of IOL [8]. A theoretical model eye study demonstrated that more than 1 mm IOL decentration and more than 5° tilt may be visually significant and cause oblique astigmatism [23]. However, according to optical simulations, considering corneal aberrations, pupil function, contrast sensitivity, and other aspects influencing the visual performance, decentration of 0.5 mm might induce significant visual symptoms [17].

Several studies have assessed the effect of IOL tilt and decentration on optical performance of eyes. This includes different types of IOL including, monofocal, spherical and aspheric, toric IOLs, and multifocal, posterior chamber as well as anterior chamber, sulcus, iris fixated and scleral fixated IOLs. In this narrative review we assess the methods of measuring IOL tilt and decentration, influence of IOL displacement on visual acuity, positive and negative dysphotopsia, and wavefront aberrations on capsular bag fixated IOLs only.

## 2. Measurement of IOL Tilt and Decentration

Methods of measuring IOL tilt and decentration have evolved in the past few decades. The employed devices include Purkinje imaging technique, Scheimpflug imaging, ultrasound biomicroscopy (UBM), and anterior segment OCT (AS-OCT). To calculate the IOL tilt and decentration a computer-assisted image processing technique is required using these devices except for CASIA2 device, a Swept Source anterior segment OCT (SS-OCT), which can automatically provide the calculation.

### 2.1. Purkinje Imaging

Purkinje imaging method has been used for a long time to measure the IOL tilt and decentration. It was presented to measure IOL tilt and decentration in 1988 in 14 pseudophakic individuals [20]. Purkinje images are reflections from the anterior (P_1_) and posterior (P_2_, usually not visible) corneal surfaces and from the anterior (P_3_) and posterior (P_4_) lens surfaces [2]. The relative positions of P_1_, P_3_, and P_4_ with respect to the centre of the pupil are detected from images. Nishi et al. [3] in 2010 reported a high intra-examiner and inter-examiner reproducibility of IOL tilt and decentration measurement using Purkinje meter with a dedicated software programme. They obtained the images subsequent to the pupil dilation and used the pupil centre as a reference point for calculation of IOL decentration [3]. Good pupil dilation is a prerequisite for the system to capture good quality images with 3 clear Purkinje reflections [3]. Janunts et al. assessed the reliability and reproducibility of purkinjemeter in pseudophakic IOL tilt and decentration using undilated pupillary centre as reference axis. They reported high reproducibility of measurements and reliability of the results. In order to further improve the reliability, they recommended taking 6 Purkinje images [24].

Previously, the radius of curvature (for the anterior surface of an IOL) was estimated by finding the best-fit sphere. Later on another method was introduced, which uses an exact ray model of imaging combined with a Zernike polynomial surface representation [25]. This method was then further extended by the same authors with particular attention to the posterior surface of the IOL in model eyes [26]. This non-contact technique is quick and easy to perform [3]. However, it depends on the radius-of-curvatures of the IOL surfaces. The third image by Purkinje meter can only show parts of the IOL anterior and posterior surfaces. This can have an effect on the radius-of-curvature and the results of IOL tilt [27]. This technique has limitations when lenses are very flat, for which P_3_ is quite large [2].

### 2.2. Scheimpflug Imaging

Scheimpflug imaging was first presented in 1989 to measure the IOL tilt and decentration [28]. In this method, following full mydriasis cross-sectional images of the anterior chamber and IOL are captured at the vertical and horizontal meridians. Then the angle of IOL tilt as well as the direction and extent of decentration will be calculated relative to a standard reference line, which connects the centre of anterior corneal surface curvature with the geometrical centre of the pupil using an image processing software [12,28]. Tilt and decentration can be obtained from Scheimpflug images using edge detection and curve fitting algorithms that allow estimation of the pupillary axis (defined as the reference) and the IOL axis [22]. De Castro et al. [2] reported high reliability with IOL tilt and measurements obtained via Scheimpflug imaging. This is a rapid method and the availability of this device may make the measurement of tilt and decentration more accessible. However, it requires more than 6 mm pupil dilation [3], which may cause the deviation of pupil centre. So the IOL decentration measured by the system should be a distance between the IOL centre and the non-real pupil axis, thus the result measured is not accurate and this method has limitations [2,29]. Also, erroneous results, often due to corneal magnification, have diminished the widespread use of the images for this purpose [30]. Powerful data processing routines, careful assessment of the limitations of the technique, and experimental validations are necessary before this information can be used reliably [2]. De Castro et al. [2] compared the Purkinje and Scheimpflug imaging methods on model eyes as well as patients. Both techniques showed high reproducibility. Optical and geometrical distortion of the Scheimpflug images produced slight discrepancies of the measured tilt and decentration, which improve with the correction of the geometrical distortion. The same authors conducted another study in 2010 [22] and validations using physical eye models indicated that Purkinje and Scheimpflug images provide estimates of IOL decentration within an accuracy of 0.026 mm and IOL tilt within an accuracy of 0.6°.

### 2.3. Ultrasound Biomicroscopy

UBM is an alternative method to measure IOL tilt and decentration. It is a contact method that generates high-resolution images of anterior segment structures [14]. Most studies measure the tilt using the angle between IOL optic plane and the posterior iris surface. Ang et al. [31] used this technique to assess axial displacement of the IOL (using the anterior chamber depth (ACD) as the reference point) as well as IOL tilt, using pupillary axis as the reference point, in 19 patients within 2 years following cataract surgery. They assessed their patients in a supine position, which may result in deepening of ACD and IOL optic tilt, so the results are not comparable with other studies on IOL tilt. However, they reported high intra-observer and inter-observer reproducibility [31]. By UBM, operators can clearly see whether the IOL has tilt and decentration, but the tilt and decentration cannot be directly measured. Moreover, it is a contact inspection method and the deformation of the eyeball extruded by water in bath cup may affect the measured results [27]. Given the above limitations and its ability to clearly depict ocular structure posterior to the pupillary plane, UBM has been more frequently used to assess the IOL stability in scleral fixated, [14,32,33,34] iris fixated [35] and sulcus IOLs [36,37] rather than a routine modality in evaluation of tilt and decentration via validated quantitative measurements. Marianelli et al. [14] used this technique to compare the IOL tilt between two groups of sutureless and sutured scleral fixated IOL groups. The mean vertical and horizontal tilts were, 0.24 ± 0.21 and 0.25 ± 0.19 mm respectively in the sutureless group and 0.14 ± 0.17 and 0.23 ± 0.16 mm respectively in the sutured group. No significant differences were seen in the vertical tilt and horizontal tilt. Zhao et al. [37] compared the sulcus fixated and capsular bag fixated IOLs tilt and decentration in children using UBM. They noticed a statistically significant difference between the groups in vertical decentration as well as the horizontal and vertical tilt. [37] They used the line between the scleral spurs as the reference line for IOL tilt measurement.

### 2.4. Anterior Segment Ocular Coherence Tomography

Anterior segment ocular coherence tomography (AS-OCT) was later employed for assessment of IOL tilt and decentration. In 2013 Wang et al. [21] reported a high inter-operator and inter-session reproducibility with this convenient, non-invasive and high-resolution imaging technique, using a 3D reconstruction method for their calculations. Like the majority of previous studies pupillary axis and pupillary plane, which was defined as the line connecting the iris smooth muscle in this paper, were their reference point to evaluate the IOL decentration and tilt. In order to minimize the potential influence of ciliary muscle accommodation on IOL tilt and decentration, they allowed 5-min dark adaptation instead of dilating the pupil with mydriatic agents. They reported tilt of 2.94 ± 0.99° and decentration of 0.32 ± 0.26 mm, 0.40 ± 0.27 mm, and 0.56 ± 0.31 mm in X, Y and spatial planes in 39 in-the-bag IOLs [21]. Another study in the same year measured the IOL tilt and decentration in 12 pseudophakic eyes, of whom 8 had congenital lens subluxation. They used 6–12 pieces of AS-OCT images based on IOL surface fitting to calculate the IOL optical axis. They reported accurate measurement of the tilt and decentration using this method [27]. Evaluation of crystalline and intraocular lens tilt and decentration with and without mydriatic drugs using CASIA2 (Tomey Corp., Nagoya, Japan) was performed by Kimura et al. [4]. It was demonstrated that both the crystalline lens and the IOL were tilted 4–6° towards the inferotemporal direction and were shifted slightly (less than 0.12 mm) towards the temporal direction. High repeatability under both non-mydriatic and mydriatic conditions were reported in this study. Moreover, significant correlations were found between pre- and postoperative data for both tilt and decentration. They used corneal topographic axis as their reference point in this study. Second generation AS-OCT is not dependent on pupil diameter, nor is it affected by pupil shape. These results are clinically significant because they showed that it is possible to assess the tilt and decentration of the crystalline lens or the IOL using second generation AS-OCT in cases of poor mydriasis [4]. Owing to its good penetration, AS-OCT has been recently used to evaluate the tilt and decentration in sulcus and scleral fixated IOLs too. The line connecting the irido-corneal scleral spurs has been the reference line to assess the IOL tilt in the majority of these studies. Kemer et al. [38] reported the mean IOL tilt angle of 3.80° ± 4.09° in the horizontal axis and 2.83° ± 4.03° in the vertical axis in 94 eyes with Z-sutured scleral fixated using AS-OCT without pupil dilation. Barca et al. [39] reported mean tilt of 2.08° ± 1.19° in 32 eyes with a sutureless single piece scleral fixated IOL following pupil dilation and Ibrahim et al. [40] reported lens decentration range between 0.21 mm and 0.9 mm and IOL tilt ranged between 1.2° and 2.8° in 6 patients with suture fixated sulcus IOLs using AS-OCT. CASIA2 (Tomey Corp., Nagoya, Japan) is a second-generation Swept source AS-OCT with scan depth of 13 mm [5]. It is an efficient method to measure IOL tilt and decentration with high repeatability. It measures the tilt and decentration, relative to the corneal vertex rather than pupillary axis. In fact compared to the pupil centre, corneal vertex is considered to be a better reference to assess tilt and decentration because it is not affected by pupil shape [11]. Sato et al. [5] assessed tilt and decentration in short-term postoperative period using CASIA2 (Tomey Corp., Nagoya, Japan) and found very high inter-observer and intra-observer reproducibility.

The other devices which use SS_OCT technology include IOLMaster 700 (Carl Zeiss Meditec, AG, Jena, Germany), Argos (Movu, Santa Clara, CA, USA) and Tomey OA-2000 (Tomey Corporation, Nagoya, Japan). These biometers have high scan depth (e.g., 44 mm in IOLMaster 700, Carl Zeiss Meditec, AG, Jena, Germany [7]) and provide high quality images at high speed. With previous methods, IOL tilt and decentration were routinely measured with reference to the iris plane or centre of the cornea. Therefore, it was impossible to be certain that the eye is measured along the visual axis. However, SS-OCT biometers offer whole-eye OCT scans and can determine crystalline lens and IOL tilt with reference to the fovea [5,7]. In fact, for IOL tilt and decentration measurements, SS-OCT has much higher repeatability than Pentacam and Purkinje meter [41]. Using IOLMaster 700 (Carl Zeiss Meditec, AG, Jena, Germany), Hirnschall et al. [6] observed the correlation between preoperative and postoperative tilt. The extent of tilt in crystalline lenses and IOLs was 4.3° ± 0.9° and 6.2° ± 1.3° respectively. They showed that before and after cataract surgery, there was, on average, a 5° outward tilt, indicating that the fovea was slightly temporal to the pupillary axis of the eye. The results in this study confirm the relative tilt of the pupillary or optical axis when the eye is aligned along the visual axis (i.e., foveal fixation). Similar study was conducted in 2019 with a higher sample size of 333 patients [7]. Both crystalline lenses and IOLs were tilted with anterior displacement of the nasal portion and there was significant mirror symmetry in tilt magnitude and direction between right eyes and left eyes. In 253 phakic and 80 pseudophakic right eyes, the mean tilt magnitude was 3.7° ± 1.1° and 4.9° ± 1.8° respectively. They found excellent repeatability for both crystalline and intraocular lenses measurement with no need to dilate the pupil. IOL tilt magnitude significantly increased compared with the preoperative crystalline lens tilt magnitude by 1.2° on average in this study. Tilt magnitude and direction of the preoperative crystalline lens and postoperative IOL were strongly correlated, indicating that preoperative phakic lens tilt could be used to predict the postoperative IOL tilt. Their findings were in agreement with Sato et al. [5], who detected inferotemporal tilt in both single-piece and 3-piece IOLs.

#### 2.4.1. Influence of IOL Tilt and Decentration on Visual Acuity

A variety of studies have assessed the effect of IOL decentration and tilt on the optical quality, mainly focusing on higher-order aberrations. However, there is a paucity of studies that are aimed at assessing the effect of decentration and tilt on near and distance visual acuity (VA). A cohort study by Madrid-Costa et al. [42] analysed differences in visual quality between two aspheric aberration-correcting and two spherical IOLs while assessing the impact of tilt and decentration on visual acuity. Visual simulation was used to evaluate ten eyes in ten patients. VA was assessed in five situations: centred lens, 0.2 mm and 0.4 mm vertical decentration, and 2° and 4° of tilt around the vertical axis. High-contrast (100%), medium-contrast (50%) and low-contrast (10%) visual acuities were measured in these five situations using the Freiburg Visual Acuity Test software. Results from the study indicated that decentration had a higher impact on VA than tilt in all types of IOLs. Overall, greater impact was seen on aberration correcting IOLs than with spherical IOLs. At high contrast (100%), decentration of all types of IOLs reduced corrected distance VA significantly. For the aspheric IOLs, mean LogMAR VA when centred was found to be −0.083 ± 0.059 and −0.04 ± 0.058 for the two types of lenses. When decentred by 0.2 mm this dropped to LogMAR 0.027 ± 0.063 and 0.083 ± 0.058 respectively, and when decentred by 0.4 mm it was LogMAR 0.124 ± 0.050 and 0.127 ± 0.045 respectively. For the spherical IOL’s, centred VA was LogMAR −0.053 ± 0.073 and −0.053 ± 0.063 respectively. When decentred by 0.2 mm this dropped to LogMAR 0.029 ± 0.049 and −0.014 ± 0.043 and when decentred by 0.4 mm it was LogMAR 0.004 ± 0.043 and 0.033 ± 0.052 respectively. When assessing IOL tilt, the researchers found statistically significant differences in visual acuity to exist with 4° tilt for both types of aspherical and one type of spherical IOLs. The results found in this simulation study were complementary to previous research, suggesting that the advantages of aspheric lenses are lost when decentration is more than 0.5 mm [2,43]. Results in this study, however, need to be taken with caution as only ten eyes were analysed.

A previous study by Hayashi et al. [1] similarly assessed near and distance VA outcomes in monofocal (MA60BM; Alcon Surgical, Fort Worth, TX, USA) and multifocal (PA154N; Allergan, Dublin, Ireland) lenses. Fifty-five eyes were used with patients being age-matched between the two groups of IOLs. The Scheimpflug video photography system was used to measure incidental decentration and tilt. Interestingly, they found no significant associations between IOL decentration or tilt and LogMAR VA at distances greater or equal to 0.7 m tested for patients implanted with the monofocal lenses. Exact figures of decentration and tilt in these lenses were not revealed in the results. On the other hand, in the multifocal group, statistically significant associations were seen between decentration equal or greater than 0.4 mm and poorer visual acuity at 5-m distance. In fact, distance VA was influenced by decentrations higher than 0.4 mm achieving a difference greater to 1 line of VA for 0.7 mm. When decentration was equal or above 0.7 mm, distance VA did not reach 0.1 LogMAR and when decentration was equal or above 0.9 mm, VA did not reach 0.2 LogMAR in any eyes. However, no significant correlation was found between the degree of tilt and the logMAR VA. The findings were similar to Madrid-Costa et al.’s [42] study summarised above, as decentration was found to once again be the major influencer on visual acuity as opposed to tilt of the lens. It has been suggested that tilt does not influence VA as long as the IOL is implanted into the capsular bag [23,44].

A more recent study from the same authors, Hayashi et al. [45] further compared multifocal (Hoya SFX MV1; Tokyo, Japan) and monofocal (VA60BBR and YA60BBR) IOLs. Forty-four eyes of 22 patients with a multifocal IOL and 44 eyes of 22 patients with a monofocal IOL were analysed. Their findings contrasted the authors’ previous findings of the effect of decentration on VA in multifocal lenses. The study found similar degrees of IOL tilt and decentration as compared to the authors’ previous work. In this study, there was no correlation between best distance-corrected VA at any distance and the degree of IOL decentration or tilt for both the monofocal and multifocal lenses. The contrasting result on this occasion was argued as being secondary to the specific Hoya refractive multifocal lens used in the study with the suggestion that different multifocal lenses would produce different results.

Fernandez et al. assessed the effect of decentration on optical function of a low-addition diffractive trifocal IOL (Alsafit Trifocal violet light filter lens, Alsanza GmbH, Germany) in an observational study [46]. They noticed greater near area under curve (NAUC) with higher decentration, which may be explained by an increase of the depth of focus due to higher order aberration induction with decentration. No specific correlation was found between decentration and intermediate or distance vision. Therefore, it was concluded that a slight decentration of such lenses may be recommendable to achieve better visual acuity at near. The same group evaluated visual performance in the presence of a decentred high-addition diffractive multifocal IOL (Bi-Flex M 677MY, Medicontur Medical Engineering Ltd. Inc., Zsámbék, Hungary) [47]. It was revealed that intermediate vision in terms of IAUC (intermediate area under curve) increases with a temporal decentration of less than 0.55 mm. This result may be justified by the location of the optical axis in reference to the fovea and neural processing rather than optical structure.

The position of the IOL in the capsular bag may change due to contraction of the bag even if the IOL has been centred during surgery. Uzel et al. [12] studied the effect of posterior capsule opacification (PCO) on IOL displacement in their retrospective study of 64 eyes. Patients with PCO were compared to controls without PCO with both groups receiving laser capsulotomy treatment. Results from the study showed that development of PCO was linked to lens displacement secondary to tilt and decentration. Nd:YAG laser capsulotomy was then found to reduce horizontal and vertical tilt, but there was no change to the decentration of the lens. No significant difference was observed in CDVA between the control group and the PCO group after capsulotomy. A significant decrease was observed in the angle of tilt at both horizontal and vertical meridians from before to after capsulotomy in the PCO group. However, there was no statistically significant difference noted between the VA of these two groups following capsulotomy.

The studies above assessing the effect of tilt and decentration on visual acuity have shown a trend of decentration having a bigger effect on outcomes than tilt of IOL. Additionally, multifocal lenses are possibly more affected by tilt and decentration than monofocal lenses. However, these studies have limited reliability as there has been no universal standard reference axis used to measure displacement. Most studies have used either the Scheimflug method or anterior segment OCT to interpret tilt and decentration. Zhang et al. [48] suggested measuring IOL displacement with reference to corneal topographic axis (CTA) to be most relevant to assessment of visual acuity postoperatively. The study found significant correlations between corrected distance VA and IOL decentration using CTA as opposed to using pupillary axis, or line of sight as references for displacement. Median corrected distance visual acuity was LogMAR 0.05 (0.00–0.22) in all patients. Mean degree of decentration was recorded to be higher in the CTA group 0.1061 ± 0.1335 mm when compared to the pupillary axis 0.0518 ± 0.1547 mm and line of sight group 0.0518 ± 0.1547 mm. Further analysis of effect of tilt and decentration on visual acuity is required using a universal reference axis to fully appreciate a trend in effect of each on visual acuity.

#### 2.4.2. Influence of Tilt and Decentration on Dysphotopsia

Tester et al. [49] first described dysphotopsia as a spectrum of undesired visual phenomenon in both phakic and psuedophakic eyes. Positive and negative dysphotopsias are accepted terms amongst clinicians and researchers to differentiate between their characteristics and presentations. The incidence of Psuedophakic dysphotopsias ranges between 33–78% in the literature [49,50,51].

Positive pseudophakic dysphotopsias are commonly elicited by a point source of light and are assumed to be caused by stray light projected onto the retina. Ray tracing models have confirmed square edge IOLs and IOLs with higher refractive index as commonest causes of positive dysphotopsia [52,53,54,55,56]. No studies to date have investigated the relationship between IOL tilt and decentration and positive dysphoyopsia. Coroneo et al. [57] demonstrated in vitro model that off-axis incident light at the temporal cornea while refracting through the nasal edge of the IOL can produce multiple secondary images and constitute glare. One can assume that horizontally displaced IOLs are more prone for gathering such intense foci in the exposed nasal edge of IOL.

Negative dysphotopsia is one of the leading causes of dissatisfaction from visual quality following uneventful cataract surgery. It usually represents with dark crescent shaped shadow in temporal peripheral visual field. Holladay et al. [58] demonstrated through ray tracing method that far peripheral retinal image beyond 80° of visual field angle is contributed by two optical paths; the rays refracted by intraocular lens itself and those missed by the IOL. The negative photopsia results from the gap between these two retinal images. They found the IOL decentration and tilt to be one of the secondary factors contributing towards this visual phenomenon alongside IOL material, diameter, edge design and aspheric surfaces. However, the primary contributory factors were observed to be smaller pupil size, larger angle kappa, a bi-convex or plano-convex IOL shape with smaller iris-to-IOL distance and anterior capsule overlying nasal IOL edge [58].

Henderson et al. [59] reviewed 59 articles in the literature to assess the aetiologies of negative dysphotopsia, and added IOL index of refraction, edge smoothness and thickness, amount of functional nasal retina, corneal shape and oedema from incision, pigmentation within the eye, prominent globe and shallow orbit as potential causes, but IOL decentration and tilt were not found to be a contributory factor.

#### 2.4.3. Influence of IOL Tilt and Decentration on Wavefront Aberrations

##### Monofocal IOLs

Monofocal IOLs are commercially available in two designs of spherical and aspherical. Spherical design comes with positive spherical aberrations. Taketani et al. [15] reported the influence of IOL decentration and tilt in 40 eyes with a spherical lens. They found that with mean IOL tilt of 3.43 ± 1.55° and the mean IOL decentration of 0.303 ± 0.168 mm, there was a significant correlation between the IOL tilt and coma-like aberrations, but no correlation was found between tilt, spherical aberrations and total higher order aberrations (HOA). They found no significant correlation between IOL decentration and coma, spherical and total HOA [15]. Thus, reducing IOL tilt decreases ocular coma aberrations and results in higher quality retinal images.

Aspherical types correct the intrinsic spherical aberration of the IOL, classified as aberration-free IOLs. The other correcting the positive spherical aberration of the average cornea; known as aberration-correcting IOLs with their asphericity calculated on average normal corneal profile in a population [42]. Aspheric IOLs have been reported to induce higher level of asymmetrical aberration when decentred or tilted.

Perez-Garcia et al. compared the effect of tilt and decentration on optical function of aspherical IOLs (with different amounts of negative spherical aberration) and spherical IOLs both theoretically (on model eyes) and experimentally (using polychromatic modulation transfer function). Aspherical IOLs were more sensitive to decentration compared to spherical IOLs. In fact, the higher quantities of spherical aberration correction were associated with a greater optical degradation. Contrarily, the effect of tilt on optical performance was less sensitive to the IOL design in the clinically typical range of tilt errors evaluated [60].

Epping et al. [61] compared different aspherical designs in a model eye study, where they found decentration leading to poor modulation transfer function (MTF) results in aberration-correcting IOLs and not so with aberration-free and spherical IOLs. At 4.5 mm pupil the dominant error was observed to be horizontal coma followed by astigmatism and defocus [61]. A prospective study on two types of aspheric implanted IOLs, AcrySof IQ IOLs (Alcon Laboratories, Fort Worth, TX, USA) in one eye and Tecnis Z9003 IOLs (AMO Inc., Santa Ana, CA, USA) in the other eye, confirmed no significant correlation between ocular HOAs and aspherical IOL tilt and decentration except for internal coma with an average tilt and decentration less than 3° and 0.3 mm, respectively [62]. In contrast, in another clinical study, Nanavaty et al. [63] found significantly more vertical coma aberrations with spherical IOLs compared to aspheric IOLs. Previous studies by Altmann et al. [43,64], Pieh et al. [65] and Tabernero et al. [30,66] have confirmed the rapid degradation of retinal image quality with tilt and decentration on wavefront correcting IOLs compared to aberration-free and spherical IOL in theoretical model eye studies. Holladay et al. [67] confirmed poor optical performance of aspheric IOLs compared to spherical IOLs with lens decentration more than 0.4 mm and tilt more than 7° in model eyes. Altmann et al. [43] demonstrated the loss of advantages offered by aspheric IOLs when decentred more than 0.5 mm. Wavefront customised IOL theoretically requires centration accuracy of 0.1 mm with a 3 mm pupil to utilise the maximum advantage offered by this IOL [68]. Similarly, in another simulated study, Dietze and Cox [69] found that the aspherical IOLs showed an increase in asymmetrical 3rd order aberrations at a faster rate than spherical IOLs when faced with increasing decentration. On the other hand, a clinical study by Mester et al. [70] found no significant difference in the correlation of wavefront aberration and lens decentration between displaced aspheric IOLs and crystalline lenses. Mckelvie et al. [71] also confirmed a trend towards increasing HOA with displacement of IOLs with greater aspheric properties. They also confirmed an established correlation between horizontal coma and vertical tilt axis in their model eye study [71]. This effect was further confirmed by Fujikado et al. [72] when comparing one spherical IOL model with three aspheric IOL models. No relationship was established between spherical aberrations and decentration and tilt, whereas vertical coma for decentred and tilted IOLs were proportional to the spherical aberration corrective power of IOLs [72]. Perez-Merino et al. [73] through laser ray tracing in another theoretical study evaluated the individual robustness of three aspheric IOL designs against their decentration and tilt to contribute to retinal image quality and HOAs. Improving surface design parameters provided better control of astigmatism and coma aberrations, with some designs (wide field aspheric optic design) more immune to optical degradation when decentred [73]. A similar correlation between decentration, tilt and HOAs in spherical and aspherical IOL designs was reported by Baumeister et al. [74] in a randomised implantation between two eyes of the same patient (*n* = 27). This study confirms no significant correlation between spherical IOLs and decentration and tilt, whereas there was a significant negative correlation between tilt and decentration in aspheric IOls. The total HOAs were not significantly correlated with tilt or decentration in either IOL model [74]. The performance of aberration-correcting IOLs is most affected by decentration followed by aberration-free IOLs and spherical IOLs.

The effect of IOL tilt and decentration on chromatic aberration correcting IOLs has not been extensively studied. Weeber et al. [75] assessed the tolerance of longitudinal spherical aberration (LSA) correcting IOLs and longitudinal chromatic aberration (LCA) correcting IOLs of model eyes to tilt and decentration against standard spherical and aspherical IOLs. They demonstrated this by decentring an aspheric refractive/diffractive IOL in four directions between 0.6 and 0.7 mm before optical performance degraded below a spherical control lens (911A, Abbott Medical Optics Inc, Santa Ana, CA, USA) and as much as 1.0 mm where performance lies below that of an aspheric IOL (Tecnis, Abbott Medical Optics Inc, Santa Ana, CA, USA); concluding the relatively good tolerance of their lenses to decentration compared to standard spherical and aspherical IOLs. Eleven percent of effective optical zone loss is estimated when a 6 mm optic IOL is decentred by 0.5 mm [76]. For a decentration greater than 1 mm or tilt greater than 5°, impairment of visual quality can occur [77].

##### Multifocal IOLs

The influence of tilt and decentration on multifocal IOLs is not consistent and uniform among available studies and this is because of variabilities of multifocal IOL designs (diffractive, refractive, hybrid, and segmental). This variability is compounded by the differences in haptic designs of these multifocal IOLs, which will have an effect on the IOL tilt and decentration. Montes-Mico et al. [78] evaluated the effect of tilt and decentration on two types of aspheric multifocal IOLs: refractive-diffractive (Acrysof AcrySof Restor SN6AD1, Alcon Laboratories Inc, Fort Worth, Texas, USA) and rotationally asymmetric segmental multifocal IOL (Lentis Mplus LS-312, Oculentis, GmbH, Berlin, Germany) in an artificial eye model. They found that rotational symmetric multifocal IOLs have higher loss of optical quality secondary to tilt and decentration compared to refractive-diffractive model, which remained stable over a range of studied tilts (2–4°) and reduced slightly with decentration (0.2–0.4 mm) [78]. Liu et al. [79] looked at the influence of tilt and decentration at two levels of decentration and two angles of tilt on a rotationally asymmetric multifocal IOL (SBL-3) in vitro. For near-horizontal (near vision zone) decentration, the optical quality was found to be better at near focus compared to distance focus. The effect was reciprocal in distance-horizontal (distance vision zone) decentration with more tendency at smaller aperture (3.00 mm) than at 4.5 mm. For near-horizontal tilting, the near focus was significantly more deteriorated than distance focus, with opposite results noted in distance-horizontal tilting with distinctive tendency at 4.5 mm aperture than 3.0 mm aperture. Contrary to other multifocal IOLs with rings, the optical quality in rotationally asymmetric segmental multifocal IOL was observed to be sensitive to decentration and tilt with better resolution at 4.5 mm aperture compared to 3.0 mm for both near and distance foci. Xu et al. [80] compared the effect of decentration on the optical quality of Tecnis mono-focal IOL (ZCB00; Abbott Medical Optics, Santa Ana, CA, USA), extended depth of focus (EDOF) Tecnis EDOF IOL (ZXR00; Abbott Medical Optics, Santa Ana, CA, USA) and bifocal (ZMB00; Abbott Medical Optics, Santa Ana, CA, USA) IOLs in a prospective non-randomised comparative study of 54 eyes. In a higher decentration subgroup (>0.25 mm), the bifocal group was reported to have significant deterioration of ocular coma aberration at 3 mm pupil diameter and internal coma at 5 mm pupil diameter alongside modulation transfer function (MTF) and point spread function (PSF) deterioration compared to the mono-focal and EDOF subgroups. A further analysis confirmed the overall IOL decentration being negatively correlated with MTF and PSF values and positively correlated with HOAs and coma aberrations only in the bifocal group.

##### Toric IOLs

IOL tilt and decentration can be affected by toricity on the IOL surface. Felipe et al. [81] evaluated the effect of toric IOL tilt and decentration on higher order aberrations in a theoretical study. They found that average modulation decay caused by the toric IOL tilt is lower than the average modulation decrement at 5° of rotation with modulation decay only noticeable in the tilt interval between 2°–5° [81]. Perez-Vives et al. [82] compared in vitro optical quality of three aspheric Toric IOLs (SN6AT3, SN6AT4, and SN6AT5; Alcon Laboratories Inc, Fort Worth, TX, USA) in relation to different degrees of decentration (diagonal, horizontal, and vertical decentring of 0.3 and 0.6 mm) at 3-mm and 5-mm pupils. They found that diagonal and vertical decentring of 0.3 mm and 0.6 mm respectively caused significant difference in vertical coma aberrations whereas diagonal and horizontal decentring of 0.3 mm and 0.6 mm respectively caused significant difference in horizontal coma aberration compared to cantered IOL positions. The increment in coma aberrations was higher in larger pupil size. These effects were assumed to be clinically negligible except when decentration reached up to 0.6 mm in vertical or horizontal meridian [82]. Nevertheless, as mentioned earlier the alignment and amount of toricity along with orientation of tilt may result in under-correction or over-correction of astigmatism in toric IOLs [16].

##### In Eyes with Previous Corneal Laser Refractive Surgery

Ruiz-Alcocar et al. [83] looked at the effect of simulated IOL tilt and decentration on spherical aberration with prior corneal laser ablation for high and low hyperopia. They assessed three different IOL designs: the Akreos Adapt (Bausch & Lomb, Rochester, New York, NY, USA), AcrySof IQ SN60WF (Alcon Lab- oratories Inc, Ft Worth, TX, USA), and Triplato Y3601075 (AJL Ophthalmic, Álava, Spain). The effect of decentration at 0.4 mm was universally poor for all three IOL types. The effect of tilt was noticed to be worse in high hyperopia ablation group compared to the low hyperopia group. Overall Akreos Adapt (aberration-free IOL) showed better immunity to adverse visual quality caused by misalignment [83]. Madrid-Costa et al. [42] compared the effect of simulated IOL tilt and decentration on spherical aberrations with prior corneal laser ablation for high and low myopia with similar adaptive optics simulator and lens models used by Ruiz-Alcocar et al. [83] In high myopia group, tilt and decentration affected the aberration-correcting IOLs more than aberration-free and spherical IOLs. Whereas the results were comparable in all three IOL models with 2° of tilt in low myopia group. There was a statistically significant differences between the aberration-free IOLs and spherical IOLs at 100%, 50%, and 10% contrast with 4° of tilt [42].

## 3. Summary

There are a few limitations in our review. This is a narrative rather than systematic review focusing on four main topics in IOL tilt and decentration, and this is because there is a paucity of randomised, controlled studies with large sample size in a prospective manner on this subject. Based on our review we propose a comprehensive study with high sample size, using SS-OCT, comparing the effect of vertical and horizontal decentration and tilt on visual acuity, refraction and astigmatism, HOA, negative and positive dysphotopsia, and patient satisfaction in the presence of different types of monofocal, multifocal, spherical, aspherical, toric, and aberration correcting IOLs.

Our review summarises, that the methods to measure the IOL tilt and decentration have significantly evolved, and the available studies have employed different methods in their measurement, while comparability of these methods is questionable. There has been no universal reference point and axis to measure the IOL tilt and decentration between different studies. The initial studies have used the pupillary axis as their reference point. This was replaced by corneal vertex in some newer studies. Subsequently, with evolution of SS OCT, the visual axis has been used as the reference point to measure the lens tilt and decentration. A remarkably high variety and brands of IOLs are used in various studies and occasionally, opposite results are noticed when two different brands of a same design were compared against another IOL design in two studies. This complicates the conclusion on the impact of tilt and decentration on different IOL type function. Low sample size is common among available studies. Also, some studies used model eyes, and some were in vivo trials, with uncertain comparability of the results.

It was noted that less than 5° of infero-temporal tilt is common in both crystalline lenses and in-the-bag IOLs with a correlation between pre-operative and postoperative lens tilt. However, the frequency of IOL tilt has been noticed to be higher in scleral fixated lenses compared with capsular fixated IOLs. The advantages of aspheric lenses are lost when decentration is more than 0.5 mm. The effect of lens displacement on visual function has been more noticeable in aberration correcting IOLs compared to spherical and standard aspherical IOLs and in multifocal IOLs versus monofocal IOLs. However, the newer IOL designs tend to have a better tolerance to tilt and decentration. Internal coma has been the most frequent type of aberration associated with IOL tilt and decentration. This increases in larger pupil size. No significant correlation has been noticed between spherical aberration and IOL tilt and decentration. IOL tilt has a minimal impact on postoperative VA. In fact, IOL decentration has a greater impact than tilt on reduction of visual acuity. Although IOL tilt produces unpredictable visual function outcome in toric IOLs, these lenses are more sensitive to rotation compared to tilt.
